# Acute Cannabis Administration Transiently Reduces Mitochondrial DNA in Young Adults: Findings from a Secondary Analysis of a Double-Blind, Placebo-Controlled, Randomized Clinical Trial

**DOI:** 10.1089/can.2023.0282

**Published:** 2024-04-02

**Authors:** Pavel Powlowski, Justin Matheson, Bernard Le Foll, Ana C. Andreazza, Ruth A. Ross

**Affiliations:** ^1^ Department of Pharmacology & Toxicology, Medical Sciences Building, University of Toronto, Toronto, Canada.; ^2^ Translational Addiction Research Laboratory, Centre for Addiction and Mental Health, Toronto, Canada.; ^3^ Institute for Mental Health Policy Research, Centre for Addiction and Mental Health, Toronto, Canada.; ^4^ Department of Family and Community Medicine, Temerty Faculty of Medicine, University of Toronto, Toronto, Canada.; ^5^ Department of Psychiatry, University of Toronto, Toronto, Ontario, Canada.; ^6^ Campbell Family Mental Health Research Institute, Centre for Addiction and Mental Health, Toronto, Canada.; ^7^ Dalla Lana School of Public Health, University of Toronto, Toronto, Canada.; ^8^ Institute of Medical Science, University of Toronto, Toronto, Canada.; ^9^ Acute Care Program, Centre for Addiction and Mental Health, Toronto, Canada.; ^10^ Waypoint Research Institute, Waypoint Centre for Mental Health Care, Penetanguishene, Canada.

## Abstract

**Background:**

Cannabis is one of the world’s most commonly used substances; however, many questions remain unanswered as to how cannabis impacts the body. Recently, there has been a resurgence of research into the effects of plant-derived cannabinoids on mitochondrial health. In particular, a number of studies implicate mitochondrial—Δ9-tetrahydrocannabinol (Δ9-THC) interactions with altered memory, metabolism, and catalepsy in mice. Although the research in this field is expanding rapidly, there is little known about the effects of cannabis on mitochondria health in human subjects either in acute or chronic term use.

**Methods:**

Blood samples were obtained from a double-blind, placebo-controlled, parallel-group randomized clinical trial in which adults who regularly use cannabis (1–4 days/week) aged 19–25 years were randomized 2:1 to receive either an active (12.5% Δ9-THC) cigarette or placebo (<0.01% Δ9-THC) cigarette containing 750 mg of cannabis before driving simulator testing. DNA was extracted from whole blood using commercial spin columns, followed by measurement of mt-ND1, mt-ND4, and β2M using quantitative polymerase chain reaction. One-way repeated measures analysis of variance (ANOVA) followed by Dunnett’s multiple comparisons test was used to observe changes in mitochondrial DNA (mtDNA) copy number over time. A two-tailed Pearsons R test was used to assess correlations between mtDNA copy number and cannabinoid levels (Δ9-THC and metabolites) in blood.

**Results:**

We found that exposure to active cannabis containing Δ9-THC, as opposed to placebo, was associated with an acute reduction in mitochondrial DNA copy number in whole blood at 15 min and 1 h after smoking. The observed decrease in mtDNA copy number negatively correlated with blood concentrations of 11-hydroxy-Δ9-tetrahydrocannabinol (11-OH-THC) and 11-Nor-9-carboxy-Δ9-tetrahydrocannabinol (THC-COOH), the two primary metabolites of Δ9-THC, but not Δ9-THC itself. Further, the negative correlation between 11-OH THC and THC-COOH concentrations and mtDNA copy number was found in only a subgroup of participants who use cannabis infrequently, suggesting a tolerance effect.

**Conclusions:**

These results illuminate mitochondrial alterations attributed to Δ9-THC consumption, which may be mediated by metabolites. These results appear to suggest stronger effects in individuals who consume cannabis less frequently, suggesting some form of tolerance to the effects of Δ9-THC and its metabolites on mtDNA content in whole blood.

**Keywords:**

Mitochondria; mtDNA; cannabis; THC; THC metabolites; blood; THC-COOH; 11-OH-THC

## Background

Cannabis is one of the most widely used psychoactive drugs worldwide. Approximately 209 million individuals reported using cannabis within the last year in 2020,^1^ with cannabis ranking as the third most commonly used controlled substance worldwide after alcohol and tobacco.^2^ The main intoxicating effects of cannabis are attributed to Δ9-tetrahydrocannabinol (Δ9-THC),^3^ which is rapidly absorbed across the mucous membranes of the lungs and is detected in the blood immediately after the first puff of cannabis.^4^ Δ9-THC exerts its principal effects through activity at cannabinoid type 1 and 2 (CB1 and CB2) receptors as a partial agonist at sub-micromolar doses.^5^ Metabolism occurs primarily in the liver by cytochrome P450 enzymes, in which the C-11 position is hydroxylated to produce 11-hydroxy-THC (11-OH-THC) and then oxidized to produce 11-Nor-9-carboxy-Δ9-tetrahydrocannabinol (THC-COOH).^6^ 11-OH-THC is psychoactive and acts as a partial agonist at the CB1 receptor with a higher binding affinity than Δ9-THC,^7^ whereas THC-COOH is considered to be non-psychoactive.^8^

Although many studies have investigated the psychotropic effects of cannabis, there remain many questions to answer. One topic that has resurged in recent years is the study of the effects Δ9-THC on mitochondrial health. Mitochondria are best known for regulating and producing the energy necessary for cellular function. However, they play multiple important roles in the body, including producing reactive oxygen species (ROS) and steroid hormones, regulating ions such as calcium, and releasing ROS and mitochondrial DNA (mtDNA), which act as signaling molecules.^9^ Although the first studies demonstrating effects of Δ9-THC on rat mitochondria were published in the 1970s,^10,11^ subsequent work has demonstrated that Δ9-THC interacting with mitochondria produces numerous significant biological outcomes. In mice, CB1-mediated Δ9-THC interactions with mitochondria regulate memory in the hippocampus,^12^ disrupt the electron transport chain in astrocytes that alters lactate-glucose metabolism,^13^ and induce catalepsy in the substantia nigra.^14^ As such, there is evidence for a significant interplay between cannabinoids and mitochondrial physiology.

In human study participants, biomarkers of mitochondrial health can be used to indirectly examine mitochondrial perturbations in response to drug treatment or disease conditions. One such biomarker is mtDNA content, measured as mtDNA copy number. Mitochondria contain a small, circular high-copy genome, which is inherited maternally and separate from the nuclear genome of the cell.^15^ Measuring mtDNA copy number in whole blood has been applied in a number of different disease conditions. Low mtDNA copy number in the periphery (whole blood) during the manic phase of bipolar disorder is associated with increased disease severity and inflammation,^16^ low mtDNA copy number is associated with increased risk of various cardiometabolic diseases,^17^ and high mtDNA copy number is associated with overall better health and survival in elderly individuals.^18^ This methodology is well suited to study a large number of biological samples and can be compared among participants across multiple timepoints after drug intervention.

In this study, we set out to investigate if there was an acute effect of cannabis administration on mtDNA copy number in whole blood from young adults who use cannabis regularly after consumption of a cannabis cigarette containing Δ9-THC, compared with a placebo with negligible Δ9-THC. We additionally wanted to assess if there was an association between physiological concentrations of Δ9-THC, or its metabolites 11-OH-THC and THC-COOH and mtDNA copy number.

## Methods

### Design and intervention

The blood samples in this study were obtained from a double-blind, placebo-controlled, parallel-group randomized clinical trial in which adults who use cannabis regularly (1–4 days/week) aged 19–25 years were randomized using a 2:1 allocation ratio to receive either a 12.5% Δ9-THC cigarette or placebo (<0.01% Δ9-THC) cigarette containing 750 mg of cannabis.^19^ Cannabidiol (CBD) content was negligible in active and placebo cannabis (<0.5%). Participants were given maximum 10 min to smoke *ad libitum* to their desired level of intoxication during the acute exposure session.

### Blood

During the acute exposure session, a registered nurse inserted an indwelling intravenous catheter for serial blood draws that occurred at baseline and at 5, 15, 30, 60, 120, 180, 240, 300, and 360 min after drug exposure. Blood was initially collected in 10 mL EDTA collection tubes and immediately transferred to cryovials, which were stored on dry ice and then frozen, initially at −20°C and then at −80°C for long-term storage. Blood samples were anonymized and referred to only by a participant ID number. DNA extraction and copy number analysis were performed blinded to the treatment condition of the participant. Sample treatment status was unblinded only during the data analysis stage. Δ9-THC, 11-OH-THC, and THC-COOH were previously quantified in each blood sample at each timepoint, as described in the original publication.

### DNA extraction

DNA was extracted from samples collected 30 min before cannabis consumption, as well as 5 min, 15 min, 30 min, and 1-h post-smoking. Blood was thawed at room temperature (21°C) for 2 h. 1:10 volume of lysis buffer (100 mM Tris-HCL, pH 8.5, 5 mM EDTA, 0.2% SDS, and 200 mM NaCl) was added to blood sample to ensure a homogenous mixing of blood, followed by mixing for 30 min on a tube rocker. DNA was extracted from samples using Blood genomicPrep Mini Spin Kit (Cytiva, England, lot#17331448) according to the manufacturer’s recommendations.

Eluted DNA concentration was quantified using Quant-IT PicoGreen™ dsDNA assay (Thermo Fisher, Waltham, MA) and diluted to 1 ng/µL for subsequent quantitative polymerase chain reaction (qPCR) analysis.

### Quantitative polymerase chain reaction

Measurements of mitochondrial gene ND1 (mt-ND1), mitochondrial gene ND4 (mt-ND4), and beta-2 microglobulin (β2M) were conducted using primers described previously by Bodenstein et al.^20^ 10 µL of SensiFAST SYBR No-ROX (Meridian Bioscience, BIO98-050, Lot #SF883-B101160) master mix was added to each well, along with 6 µL of extracted DNA diluted to 1 ng/µL, 1 µL of each forward and reverse primer per gene at a concentration of 10 µM, and 2 µL of water. Samples were loaded in technical triplicates into 384-well PCR plates (FroggaBio, 4TI-1387). Each plate contained no template controls for all three genes. Additionally, each plate contained measurements of all three genes in a reference sample consisting of a combination of all extracted DNA to ensure correct master mix preparation and verify inter-plate variability. PCR was conducted using a CFX384 real-time PCR system (BioRad, Hercules, CA). Samples were heated to 95°C for 3 min, followed by 95°C for 10 sec, 60°C for 20 sec, and a plate-read, cycled 40 times. Sample quantification cycle (Cq) values were extracted using BioRad CFX Manager.

### Data analysis

Standard deviation was calculated within each technical triplicate, and Cq values that were greater than one standard deviation from the mean were eliminated from analysis. The remaining Cq values for each gene measured in each sample were averaged, and the ΔCt value was calculated for ND1 and ND4 (Cq_β2M_-Cq_ND1/ND4_). Copy number was measured defined as 2^-ΔCt^.

Sample treatment groups were then unblinded, and mtDNA copy number was plotted in GraphPad Prism 6 (La Jolla, CA) according to the treatment group. All further data analysis was conducted using GraphPad Prism 6. mtDNA copy number outliers were assessed within each timepoint for each treatment condition and removed from further analysis using Rout (Q = 1.0%). If one timepoint was eliminated for a participant because of outlying mtDNA copy number, the participant’s data were entirely removed from further analysis to be able to run repeated-measures ANOVA statistical tests; column means were not used to replace missing values. Data normality was assessed using D’Agostino & Pearson omnibus normality test. ND1 content followed a normal distribution at all timepoints in the placebo and active treatment groups, whereas ND4 content was not normally distributed at all timepoints. A two-tailed, independent samples *t*-test was used to compare age and body mass index (BMI) of the placebo and active cannabis treatment groups. A two-tailed chi-squared test was used to compare the proportion of tobacco smokers as well as males and females in the placebo and active cannabis groups. A Mann–Whitney *U* test was used to compare cannabinoid dosages in males and females. An unpaired *t-*test was used to compare differences in basal mtDNA levels between males and females, as well as low- and high-frequency smokers. Repeated-measures ANOVA using the Geisser-Greenhouse correction, followed by Dunnett’s multiple comparisons test, was used to test if mtDNA copy number changed over time relative to the baseline measurement. Analysis of correlations between mt-ND1 and mt-ND4 copy number measurements, as well as mtDNA copy number and Δ9-THC, 11-OH-THC, and THC-COOH levels, were conducted using a two-tailed Pearson’s R test. Further correlations between 11-OH-THC and THC-COOH and Δ9-THC were made by separating the active treatment group into high-level and low-level metabolite groups by the median split of the measured blood metabolite levels at 5 min post-smoking, or by splitting the active cannabis treatment group by the median number of self-reported days of smoking per week.

## Results

### Participant characteristics

Demographics of the study population after removal of mtDNA outliers (Table 1) was used to assess if there were any significant differences in age, BMI, or smoking status. Importantly, in this study cohort, there was no significant difference in the age (*t*[75] = 0.0000, P > 0.9999], BMI (*t*[75] = 0.0089, *p* = 0.9929), or tobacco smoking status (χ^2^[1] = 0.3810, *p* = 0.5371) of the participants in the placebo group and the active cannabis group. While sex can also have an impact on mtDNA copy number, there was no significant difference in the proportion of sexes in the placebo and active treatments (χ^2^[1] = 0.07903, *p* = 0.7786). Males and females did not differ in the concentration of 11-OH-THC or THC-COOH at any of the timepoints measured, whereas Δ9-THC levels were higher in males at 1-h post-smoking *p* < 0.05), (Supplementary Fig. S1A). Furthermore, baseline mtND-1 and mt-ND4 copy number did not differ in males and females (Supplementary Fig. S1B).

**Table 1.** Study Demographics of Participants Included in Final mitochondrial DNA Copy Number Analysis

Study demographics	
Participants per group	
Placebo (<0.01% Δ9-THC)	25
Active Cannabis (12.5% Δ9-THC)	52
Sex	
Male	57
Placebo	18
Active Cannabis	39
Female	20
Placebo	7
Active Cannabis	13
Age	
Placebo	22 ± 2.219
Active Cannabis	22 ± 1.885
BMI	
Placebo	24.65 ± 4.212
Active Cannabis	24.64 ± 4.776
Tobacco smoking status (smokers vs. non-smokers)	
Placebo	32.00%
Active Cannabis	28.85%
Daily smokers (daily vs. occasional + non-smokers)	
Placebo	12.00%
Active Cannabis	11.52%

Δ9-THC, Δ9-tetrahydrocannabinol.

### mtDNA copy number after cannabis exposure

To ensure that mtDNA was selectively measured during qPCR, we measured copy numbers for mt-ND1 and mt-ND4 (located on opposite sides of the mtDNA genome). mt-ND1 and mt-ND4 copy numbers from all amplifications were correlated using a two-tailed Pearson’s R test (Supplementary Fig. S2) and demonstrated a very strong correlation (r = 0.9488, *p* < 0.0001, *n* = 449 samples). These results indicate that mtDNA was successfully amplified in these blood samples, rather than off-target replication during qPCR. The time course of measured mtDNA copy number for placebo and active cannabis treatment groups was plotted separately (Fig. 1). Although there was no significant change observed over time in the placebo cannabis treatment group, *F*(3.093,74.23) = 2.004, *p* = 0.1188, mtDNA significantly changed over time in the active group, *F*(3.718,189.6) = 3.801, *p* = 0.0066. Compared with baseline, mtDNA copy number was significantly lower at 15 min (2.667, *p* < 0.05) and at 1 h (2.750, *p* < 0.05) post-smoking. Sex disaggregated results for female and male active treatment group study participants are presented in Supplementary Figure S1A; however, the results of this subgroup analysis should be interpreted with caution because of the small sample size of females.

**FIG. 1.**
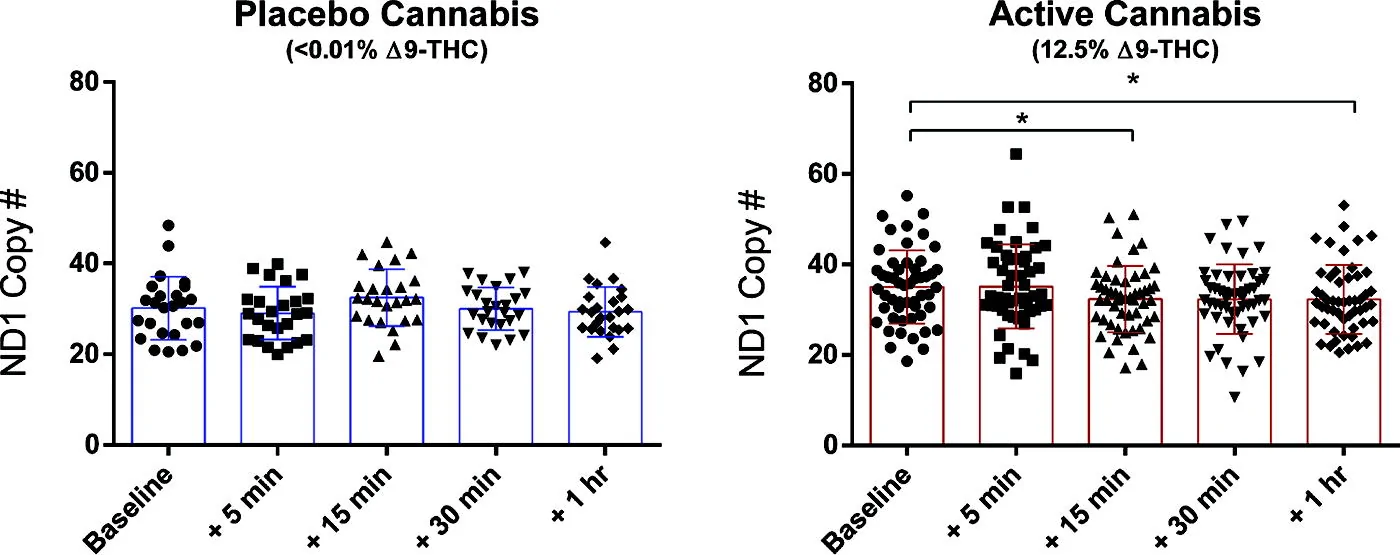
Active cannabis participant group demonstrates a decreased mtDNA copy number. mtDNA copy number was plotted for all timepoints assessed in placebo and active cannabis treatment groups separately. A repeated-measures ANOVA followed by Dunnett’s multiple comparisons test was used to test differences in mtDNA copy number post-smoking as compared with baseline levels (30 min before consumption of the cannabis cigarette). In the active cannabis group, mtDNA is significantly lower at 15 min and 1-h post-smoking. * = *p* < 0.05. ANOVA, analysis of variance; mtDNA, mitochondrial DNA.

### Correlations between cannabinoids and mtDNA copy number in blood

In order to test if there was an association between the levels of Δ9-THC, 11-OH-THC, or THC-COOH, the primary metabolites of Δ9-THC, correlations between mtDNA copy number, and measured blood levels of Δ9-THC and its metabolites were tested (Fig. 2). Although there was no correlation between Δ9-THC levels and mtDNA copy number, there was a negative correlation observed between 11-OH-THC and mtDNA copy number (ND1 r = −0.1658, *p* = 0.0074, ND4 r = −0.1759, *p* = 0.0044, *n* = 260 samples) and THC-COOH and mtDNA copy number (ND1 r = −0.1716, *p* = 0.0055, ND4 r = −0.1780, *p* = 0.0040, *n* = 260 samples). Although these results were intriguing, the correlations observed had small effect sizes. Therefore, participants in the active cannabis group were stratified into two groups based on the median blood concentration of 11-OH-THC, THC-COOH, or Δ9-THC at 5 min post-smoking, and correlations between mtDNA copy number and the correlations were tested again. mtDNA correlated negatively with 11-OH-THC (ND1 r = −0.1964, *p* = 0.0251; ND4 r = −0.2091, *p* = 0.0170, *n* = 130 samples) and THC-COOH (ND1 r = −0.2275, *p* = 0.0092; ND4 r = −0.2497, *p* = 0.0042, *n* = 130 samples) in the high blood concentration participant groups (Fig. 3A), whereas neither of the low concentration metabolite groups demonstrated any correlation between mtDNA copy number and metabolite level in the blood (Supplementary Fig. S3B). There was no correlation between Δ9-THC levels and mtDNA copy number in high or low concentration Δ9-THC groups (Supplementary Fig. S4A). Separating the active treatment participant group from the same study sample by the median number of days per week of self-reported cannabis smoking (median 2.5 days), correlations between blood 11-OH-THC or THC-COOH and mtDNA were assessed in individuals who smoked 1–2.5 days/week and 3–4 days/week. In this correlation analysis, it was observed that the negative correlation between mtDNA and 11-OH-THC (ND1 r = −0.3134, *p* = 0.0002, ND4 r = −0.3272, *p* = 0.0001, *n* = 135 samples) and THC-COOH (ND1 r = −0.3380, *p* < 0.0001, ND4 r = −0.3232, *p* = 0.0001, *n* = 135 samples) was driven by the low usage group of individuals who smoked 1–2.5 days/week (Fig. 3B), and there was no correlation observed between blood metabolite concentration and mtDNA copy number in the high-frequency smoking group (SupplementaryFig. S3C). Importantly, there was no difference in baseline mt-ND1 or mt-ND4 levels in the low- and high-frequency smoking groups (Supplementary Fig. S1B). There was no correlation between Δ9-THC concentration and mtDNA levels in either usage group (Supplementary Fig. S4B).

**FIG. 2.**
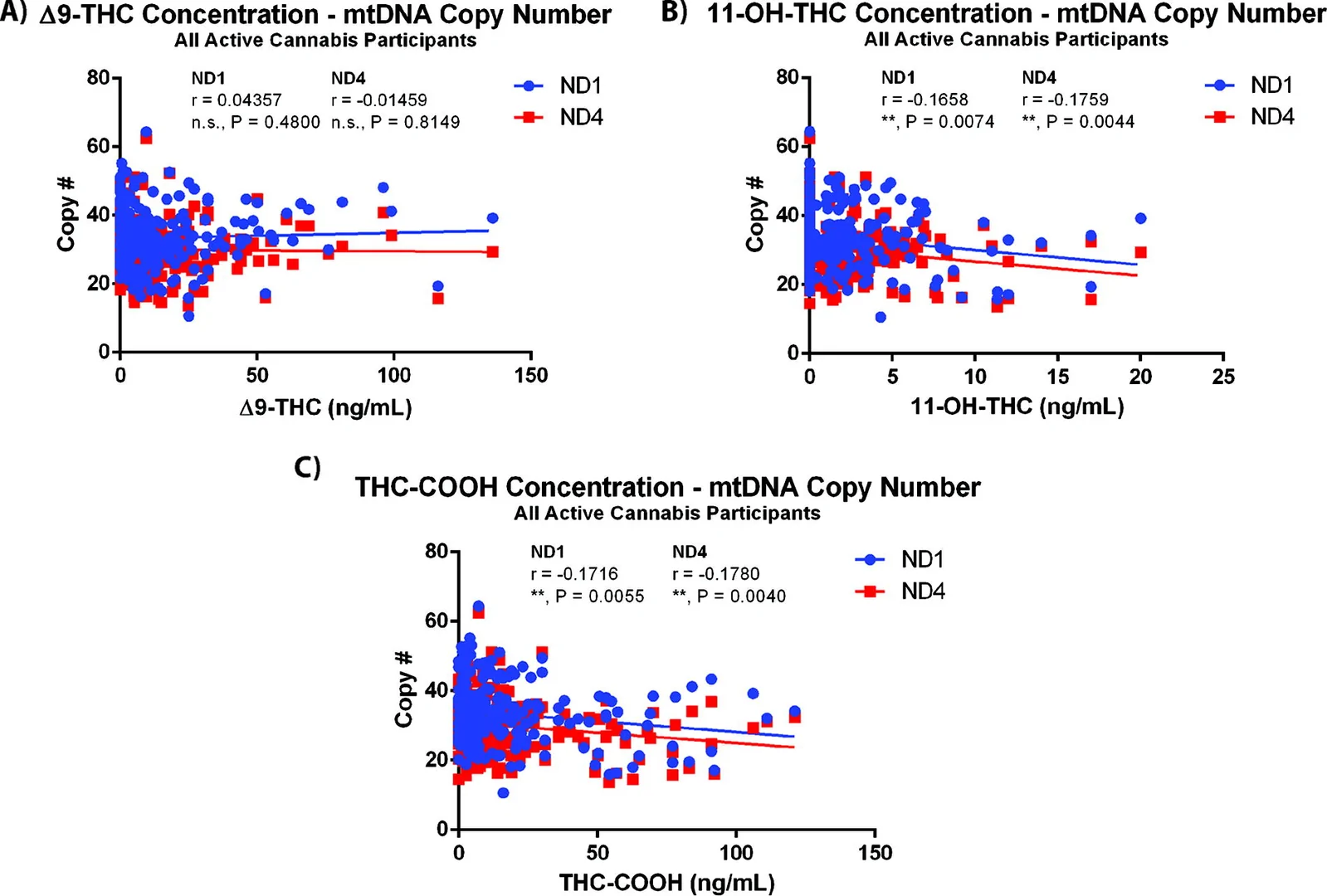
mtDNA copy number negatively correlates with Δ9-THC metabolite levels in blood, but not with Δ9-THC levels. Correlations between mtDNA copy number and the measured doses of **(A)** Δ9-THC, **(B)** 11-OH-THC, **C)** THC-COOH. Correlations were measured using a two-tailed Pearson’s R test. r values and P for each metabolite are reported in the corresponding graph, * = *p* < 0.05, ** = *p* < 0.01. mtDNA, mitochondrial DNA; Δ9-THC, Δ9-tetrahydrocannabinol; 11-OH-THC 11-hydroxy-Δ9-tetrahydrocannabinol; THC-COOH, 11-Nor-9-carboxy-Δ9-tetrahydrocannabinol.

**FIG. 3.**
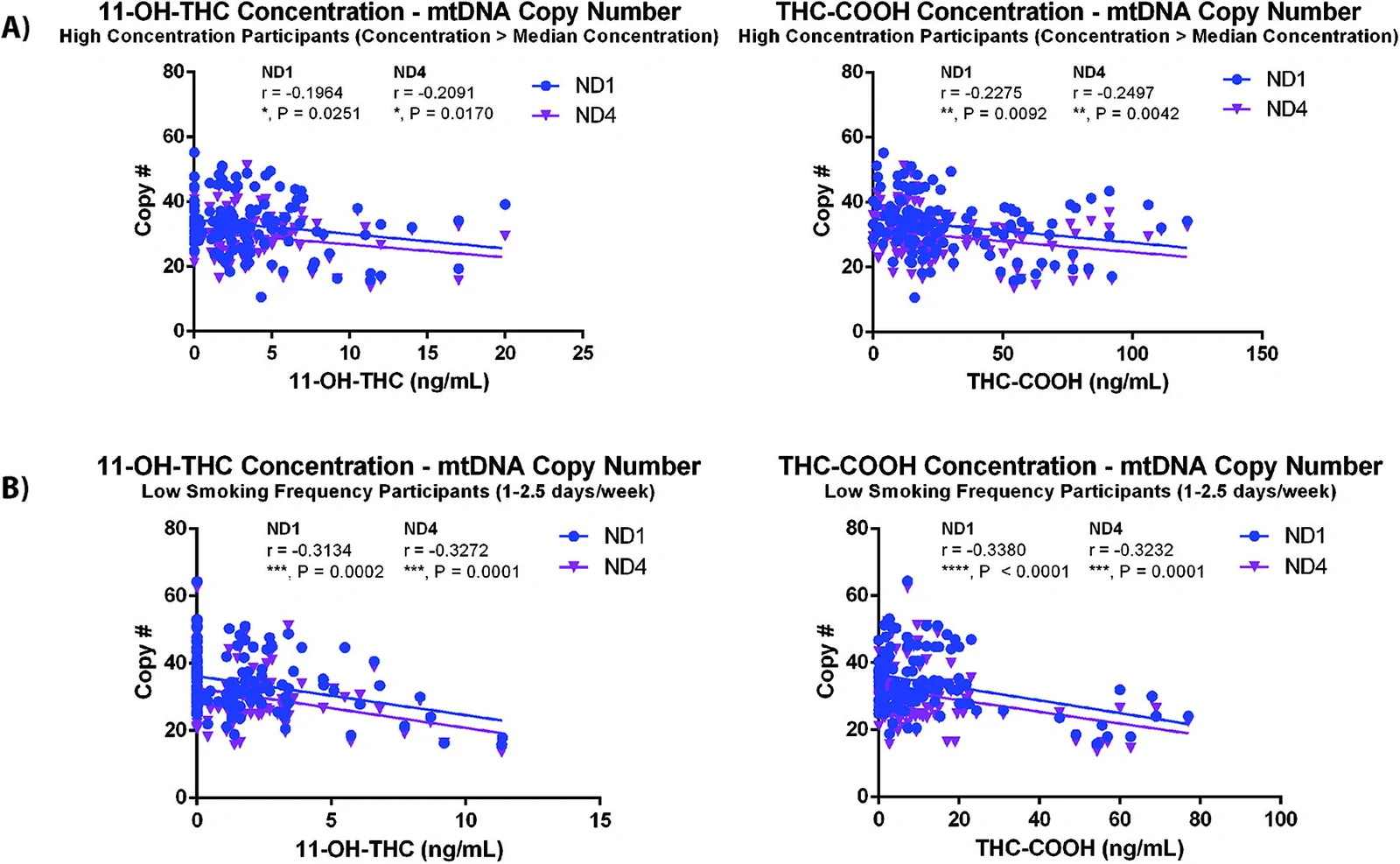
mtDNA copy number correlation strength improves with separation of active cannabis participant group by median blood metabolite concentrations and self-reported smoking frequency (days/week). Correlations between mtDNA copy number and the measured doses of 11-OH-THC and THC-COOH stratified by **(A)** median blood metabolite concentrations at 5 min post-smoking, **(B)** self-reported number of days of smoking per week. The participants shown had a median 11-OH-THC concentration above 2.30 ng/mL, and THC-COOH was above 8.40 ng/mL. The median number of days reported of smoking per week was 2.5, and participants shown smoked 1–2.5 days/week. Correlations were measured using a two-tailed Pearson’s R test. r values and P for each metabolite are reported in the corresponding graph, * = *p* < 0.05, ** = *p* < 0.01., *** = *p* < 0.001, **** = *p* < 0.0001. mtDNA, mitochondrial DNA; 11-OH-THC, 11-hydroxy-Δ9-tetrahydrocannabinol; THC-COOH, 11-Nor-9-carboxy-Δ9-tetrahydrocannabinol.

## Discussion

In this study, we found that acute exposure to cannabis containing Δ9-THC was associated with reductions in mitochondrial DNA in whole blood 15 min and 1 h after smoking cannabis under laboratory conditions. Correlations with blood levels of Δ9-THC or its metabolites suggest that this effect is associated with the metabolites of Δ9-THC, 11-OH-THC, and THC-COOH, rather than Δ9-THC itself. Separation of the active treatment group into high and low blood metabolite levels suggests that this is a concentration-dependent effect. Interestingly, the negative correlation observed between higher concentrations of 11-OH-THC and THC-COOH with mtDNA copy number was observed in participants who reported less frequent cannabis use (1–2.5 days/week), but not in participants reporting more frequent use (3–4 days/week). Taken together, our results provide the first evidence from a placebo-controlled human laboratory study that acute exposure to smoked cannabis may cause reductions in mtDNA.

One molecular candidate of interest in the context of altered mtDNA content after cannabis consumption is ROS. Treatment of isolated rat brain mitochondria with Δ9-THC increases hydrogen peroxide production and free radical leak from mitochondria,^21^ although these effects were not proven to be CB1 dependent. In coronary endothelial cells, activation of the CB1 receptor by the endocannabinoid anandamide or synthetic agonist HU210 for 3 h is associated with the production of ROS.^22^ Exposure of cells *in vitro* to hydrogen peroxide has been associated with reduced mtDNA amplification,^23^ which is accompanied by an increase in the amount of linear mtDNA within 1 h of exposure to hydrogen peroxide and, subsequently, results in mtDNA degradation.^24^ Interestingly, Jimenez-Blasco et al.^13^ demonstrate a CB1-dependent decrease in ROS in primary mouse astrocytes after a 24-h treatment with THC or HU210, which suggest that these effects are cell-type dependent or treatment duration dependent.

With regard to the effects of Δ9-THC metabolites, potential mechanisms are more elusive. A number of different biological roles for THC-COOH have been suggested, including inhibition of platelet-activating factor activity,^25^ inhibition of ALOX15,^26^ and prostaglandin synthesis.^27^ However, conclusive evidence with regard to how these signaling modalities impact mtDNA content in whole blood is not available. Interpreting these effects of THC-COOH in the context of mtDNA content is further compounded by the proinflammatory activity of smoke inhalation.

These results additionally suggest that there is a potential effect of tolerance to exposure to Δ9-THC and its metabolites. Although the measured blood concentration range of both metabolites was similar in the low- and high-frequency usage groups, there was no correlation between concentration and mtDNA copy number in the high-frequency usage group. Regular consumption of cannabis containing Δ9-THC has been demonstrated to produce a rapid tolerance, as well as reduced behavioral responses to cannabis consumption.^28^ Those effects are associated with adaptation of the endocannabinoid system such as downregulation of the CB1 receptor^29^ and also of fatty acid amide hydrolase.^30^ Although the effects of Δ9-THC and its metabolites on mtDNA copy number have not been shown here to depend on CB1 receptor activation, more frequent cannabis use may cause downregulation of the molecular targets of THC and its metabolites that induce the alterations to mtDNA copy number observed.

One consideration that must be made in the context of these findings is the heterogeneous composition of whole blood. A major limitation of this secondary analysis is that the blood samples were frozen and thus precluded counting blood cell types present in samples at each timepoint. Very broadly, whole blood contains red blood cells (erythrocytes), platelets, white blood cells, and plasma,^31^ which circulates through vasculature lined by a monolayer of endothelial cells.^32^ In mammals, red blood cells contain no mitochondria or nucleus,^33^ whereas white blood cells contain both a nucleus and mitochondria, and platelets contain mitochondria and no nucleus.^34^ It is, therefore, possible that changes to mtDNA content in whole blood reflect changes to the number of platelets in blood.^34^ Exposure of red blood cells *in vitro* to 24 µg/mL of a cannabinoid mixture containing 8 common cannabinoids at equal doses increased hemolysis and free hemoglobin levels at all tested timepoints.^35^ Exposure of platelets to the same cannabinoid mix reduced platelet counts, ATP content, and platelet aggregation. This suggests that some of the variations in mtDNA content observed may be because of changes to platelet counts; however, our findings also suggest that these changes are limited by some form of tolerance. Subsequent studies should evaluate changes to blood cell counts after cannabis consumption, or evaluate cell-free mtDNA, which would eliminate issues related to blood cell heterogeneity.

## Conclusions

We report here, to the best of our knowledge, the first evidence from a placebo-controlled human laboratory study that cannabis containing Δ9-THC is associated with altered mitochondrial physiology in young, healthy adults. These results suggest that there is an aspect of tolerance with regard to the observed effects on mitochondria. Further work should investigate the molecular mechanisms underlying these observed changes and elucidate which cell types are affected by the consumption of Δ9-THC. Currently, it remains difficult to determine if these effects of Δ9-THC and its metabolites on mitochondria are harmful to blood cells/vasculature; however, they should be considered in the context of determining how cannabis use impacts blood and the cardiovascular system.

## Data Availability

Data can be made available upon reasonable request to the authors.

## Notes

### Abbreviations Used

11-OH-THC: 11-hydroxy-Δ9-tetrahydrocannabinol
Δ9-THC: Δ9-tetrahydrocannabinol
ALOX15: 15-lipoxygenase
BMI: body mass index
CB1: cannabinoid type 1 receptor
CB2: cannabinoid type 2 receptor
MtDNA: mitochondrial DNA
Qpcr: quantitative polymerase chain reaction
ROS: reactive oxygen species
TCY: thrombocytopenia
THC-COOH: 11-Nor-9-carboxy-Δ9-tetrahydrocannabinol
